# Investigating the effectiveness and feasibility of exercise on microvascular reactivity and quality of life in systemic sclerosis patients: study protocol for a feasibility study

**DOI:** 10.1186/s13063-018-2980-1

**Published:** 2018-11-21

**Authors:** Alexandros Mitropoulos, Anil Gumber, Helen Crank, Mohammed Akil, Markos Klonizakis

**Affiliations:** 1 Centre for Sport and Exercise Science, Sheffield Hallam University, Sheffield, S10 2BP UK; 2 Centre for Health and Social Care Research, Sheffield Hallam University, Sheffield, UK; 3 Rheumatology Department, Royal Hallamshire Hospital, Sheffield, UK

**Keywords:** High-intensity interval training, resistance training, vascular function, quality of life, digital ischemia, feasibility studies

## Abstract

**Background:**

Raynaud’s phenomenon is one of the first clinical manifestations observed in systemic sclerosis (SSc). This microvasculature disorder affects mostly the digits in over 95% of SSc patients, significantly affecting their health-related quality of life (HRQoL) and incurring higher hospital admissions and other healthcare costs. Exercise is known to improve both micro- and macrovascular function – aerobic exercise and resistance training, separately or combined, have been demonstrated to lead to significant vasculo-physiological improvements in conditions that present vasculopathy. However, the effects of a combined exercise programme on microcirculation in SSc patients has yet to be investigated. Therefore, the purpose of this study is to assess the effects of high-intensity interval training (HIIT) combined with circuit resistance training on the microvascular function in the digital area of SSc patients.

**Methods:**

This will be a randomised controlled, feasibility trial with two arms, wherein 30 patients with SSc in receipt of medical treatment will be randomly assigned to usual care (medical treatment) or to a 12-week supervised exercise programme. Patients in the exercise group will undertake two, 45-min sessions each week consisting of 30 min HIIT (30 s 100% peak power output/30 s passive recovery) on the arm crank ergometer and 15 min of upper body circuit resistance training. Patients will be assessed before as well as at 3 and 6 months following randomisation. Primary outcomes of the study will be recruitment and retention rate, intervention acceptability and adherence to the exercise programme. Secondary outcomes include the digital area cutaneous microvascular function (laser Doppler fluximetry combined with iontophoresis), physical fitness, functional ability, upper back transcutaneous oxygen tension, body composition and quality of life (EQ-5D-5L). Selected interviews with a subsample of patients will be undertaken to explore their experiences of having Raynaud’s phenomenon and the acceptability of the exercise intervention and study procedures.

**Discussion:**

Data from this study will be used to identify the feasibility of a combined exercise programme to be implemented in SSc patients, the acceptability of the intervention and the study design, and to determine the effects of exercise on the microvasculature. Overall, this study will provide sufficient data to inform and support a full multicentre clinical trial.

**Trial registration:**

ClinicalTrials.gov (NCT number): NCT03058887, February 23, 2017.

**Electronic supplementary material:**

The online version of this article (10.1186/s13063-018-2980-1) contains supplementary material, which is available to authorized users.

## Background

Systemic sclerosis (SSc) is an autoimmune connective tissue disease characterised by tissue fibrosis and vascular involvement. Vasculopathy is directly implicated in the pathogenesis of SSc representing acute manifestations (e.g. pulmonary arterial hypertension, digital ulcers). Raynaud’s phenomenon (RP) is one of the first clinical manifestations presented in SSc, with a 95% prevalence across patients. RP precedes any other clinical symptoms of SSc and is due to hypoxia in the extremities in response to cold. Evidence suggests that endothelial injuries in conjunction with an imbalance in the vascular tone trigger RP [1], which in turn develops digital ischemia, a serious complication for patients with SSc. Morbidity rates in patients with SSc due to digital ischemia reach approximately 30% annually [2]. Patients with chronic digital ulcers develop irreversible tissue loss [2], which usually requires hospitalisation for iloprost infusion [3] lasting from 5 days to 3 weeks. Hospitalisation is a psychologically stressful procedure for the patient, directly affecting their health-related quality of life (HRQoL). The most common side effects of iloprost infusion are headache, flushing of the skin, nausea, vomiting and sweating. Amputation has been reported to occur in one or more digits due to ischemia in 20.4% of patients with SSc, 9.2% of which have multiple digit loss [4].

A recent review paper has aggregated the studies that have examined the effects of exercise in SSc patients with and without pulmonary involvement [5], summarising the beneficial effects, including good exercise tolerance and increased aerobic capacity [6–8], muscle strength [6, 9], hand mobility, [10–12], functional ability in daily activities [11] and increased HRQoL [10, 11]. However, most studies lacked reporting of the intensity of their exercise programme and none examined the effects of exercise on the vasculature. Therefore, it is important to explore the feasibility of a training protocol that would be able to (1) improve vascular function and potentially decrease the frequency and severity of digital ischemia and ulcers, and (2) have the potential to decrease the use of pharmacological agents. These agents are used to improve microcirculation with short-term (e.g. oedema, headaches, heart palpitations, dizziness and constipation) and long-term (e.g. heart dysfunction, increased cardiovascular risk) side effects and financial cost implications.

High-intensity interval training (HIIT) has demonstrated its efficacy in improving the endothelial function in the macrovasculature compared to moderate intensity continuous training in a range of clinical conditions with impaired vascular function [12]. HIIT has been shown to improve cardiorespiratory fitness, cardiovascular risk factors and biomarkers associated with vascular function. A HIIT protocol with short intervals (30 s exercise/30 s passive recovery; no exercise during resting intervals) may elicit more favourable patient-reported satisfaction/enjoyment levels compared to other longer duration exercise protocols [13]. In chronic heart failure patients, a short duration HIIT protocol (30 s exercise/30 s passive recovery) was demonstrated to be well tolerated, a preferred protocol with a low perception of effort, greater patient comfort and with a longer time spent at higher percentage of peak oxygen uptake (VO_2peak_) than a longer duration HIIT protocol with active recovery phases (low intensity exercise during resting intervals) [13].

Resistance training (RT) alone has been demonstrated to be effective in improving the endothelial vascular function in obese [14] and type 2 diabetes mellitus patients [15] where a vascular dysfunction is present in the pathophysiology of these conditions. The intensity of the RT ranged from 75 to 85% of one repetition maximum for 8 to 12 weeks (twice or thrice per week). However, a combined exercise regime (aerobic and RT) has also shown significant improvements in endothelial vascular function in a range of clinical conditions. More specifically, Metsios et al. [16] examined the effects of a combined exercise protocol in rheumatoid arthritis patients who performed a 6-month exercise intervention. Endothelial function was assessed via laser Doppler fluximetry (LDF) imaging using iontophoresis. The results indicated that an individualised aerobic and strength training programme significantly improves both micro- and macrovascular endothelial function in patients with rheumatoid arthritis. Other studies that have implemented a combined exercise protocol in chronic heart failure [17] and type 2 diabetes mellitus patients [18] have demonstrated a significant improvement in the microvascular reactivity of the forearm conduit and resistance vessels after an 8-week programme. Consequently, it seems that either RT alone or a combined protocol are able to improve endothelial vascular function.

To our knowledge, the feasibility of a combined HIIT protocol with short intervals (30 s at 100% peak power output and 30 s passive recovery) and a circuit RT programme on SSc patients has not yet been examined. Moreover, the efficacy of exercise on microcirculation in SSc patients constitutes another unexplored element of SS. Therefore, the primary aim of our study would be to investigate the feasibility of exercise in patients with SSc and the secondary aim is to explore the effects of exercise on microcirculation. Our study objectives are (1) to estimate the rates of recruitment and retention for a future definitive trial of a 12-week supervised exercise programme for SSc patients; (2) to estimate the rates of attendance to and compliance with the supervised exercise programme; (3) to assess the tolerance, acceptability and enjoyment levels of the training protocol; (4) to investigate the effects of exercise on microvascular reactivity in the digital area; (5) to identify and quantify the training dose that may induce microcirculatory improvements in SSc patients; (6) to assess the effects of exercise on HRQoL of SSc patients measured through generic questionnaires and interviews; and (7) to conduct post-intervention interviews with patients to explore individual’s experiences of the study’s procedures and exercise programme.

## Methods/Design

### Study design

**Fig. 1:**
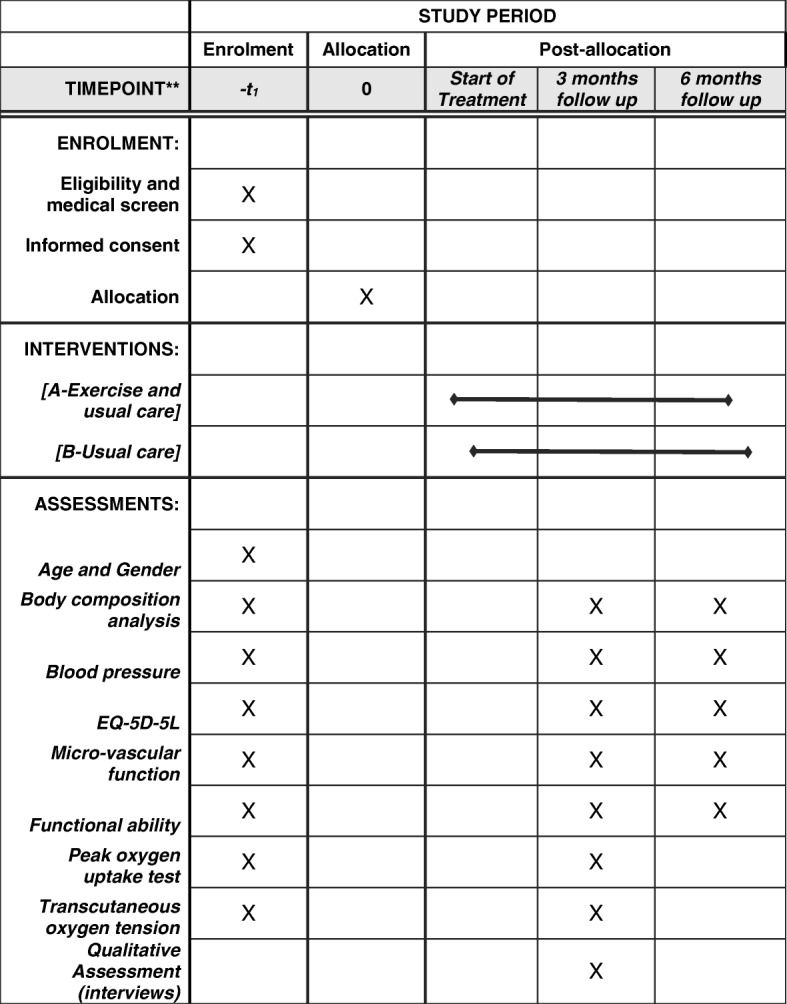
Study design

The FESS (Feasibility of Exercise in Systemic Sclerosis patients) study is a mixed methods feasibility study involving a randomised controlled trial. Thirty patients with SSc receiving usual care (e.g. medical treatment) will be randomly assigned (via block randomisation remotely by the study chief investigator) to either an exercise group (12-week supervised exercise programme) or to a control group being in receipt only of the usual care. Patients will be followed for 6 months following randomisation (Fig. 1). It is not feasible to perform masking of patients or study investigators regarding the allocated treatments due to limited resources. The study is being run at Sheffield Hallam University in collaboration with the Royal Hallamshire Hospital (Sheffield Teaching Hospitals, NHS, UK). The study’s protocol and registration have been published in ClinicalTrials.gov (NCT number: NCT03058887).

This study started in February 2017 and is due to be completed by May 2018. The trial is sponsored by the Vice Chancellor’s Fellowship award of Sheffield Hallam University, and ethical approval was granted by the NHS Health Research Authority, London Committee (16/LO/0811), which allows ethical permission across Sheffield Hallam University and Royal Hallamshire Hospital (Sheffield Teaching Hospitals).

### Recruitment of participants

Patients conforming to the eligibility criteria will be recruited from the Rheumatology Department of the Royal Hallamshire Hospital in Sheffield or will be self-referred by seeing a poster in this location, through responding to a public announcement, or after being notified of the study via word of mouth. Patients recruited via hospital clinics will be invited for participation through an invitation letter (including the contact details of a research team member) accompanied by a patient information sheet. Patients who express an interest (calling or posting back) will be contacted by the research team to confirm their eligibility, addressing any inquiries regarding the study and arranging the first visit. Clinical care will not be affected for those not wishing to take part in the study nor those who decide to withdraw from the study.

The first visit will take place at the Centre for Sports and Exercise Science at Sheffield Hallam University laboratories, where the patient will be given further details of the study procedures and will be asked to sign a consent form to participate in the research study. Afterwards, patients will be habituated with the trial processes and will complete the baseline assessments.

### Eligibility criteria

Patients eligible for the trial must comply with all of the following at randomisation:

#### Inclusion criteria

- ➢ Patients diagnosed with limited cutaneous systemic sclerosis according to the 2013 ACR/EULAR criteria experiencing RP
- ➢ Men or women aged 18–80 years old
- ➢ Disease duration between 1 and 10 years
- ➢ Patients should be able to perform exercise

#### Exclusion criteria

- ➢ Patients with advanced pulmonary arterial hypertension or interstitial lung disease
- ➢ Patients diagnosed with another inflammatory condition
- ➢ Patients presenting myositis with proximal muscle weakness
- ➢ Patients with New York Heart Association class 3 or 4
- ➢ Current smokers or people who stopped smoking within 4 weeks of health screening
- ➢ Women who are currently pregnant

#### Withdrawals

Patients will be considered as withdrawn from the research trial if they request to leave the trial, if they are lost to follow-up or if they die before completing the 6-month follow-up.

### Baseline measurements

At Visit 1, following the provision of written informed consent and the confirmation of eligibility (which will include a medical examination), the following baseline measurements will be recorded:

- Demographic data, including age, sex and socioeconomic status
- Clinical history, including incident or recurrent RP, duration of disease, duration of RP attacks, current medications, height, weight, body composition (percentage body fat and lean muscle mass)
- Baseline exercise history
- HRQoL questionnaires: EQ-5D-5L [19] and RP diary and condition score [20]
- Cutaneous microvascular function using LDF combined with the iontophoresis technique in the reference finger [21, 22]
- Functional capacity using the 6-minute walking test [23]
- VO_2peak_ will be performed on an arm-crank ergometer

### Exercise intervention

The exercise group will undertake twice weekly supervised exercise sessions at the Centre of Sport and Exercise Science at Sheffield Hallam University for 12 weeks (total of 24 sessions). The weekly training structure and the training hours will be flexible to work around patients’ commitments, although sessions in two consecutive days will be avoided to allow sufficient recovery between sessions. The duration of the exercise intervention is based upon evidence reporting that an average training dose–response of 12 weeks significantly improves the macrovasculature in several disease states [12]. A maximum of 14 weeks will be allowed for the patients to complete the 24 sessions, to allow for missed sessions due to, for example, illness or holiday.

Each session will comprise of a 5-min warm-up period (involving light aerobic exercise and gentle range of motion exercises). This will be followed by an arm-cranking HIIT for 30 s at 100% of peak power output interspersed by 30 s passive recovery for a total of 30 min. The HIIT protocol will be combined with RT lasting for a total of 15 min. RT will consist of an upper body circuit training (five exercises) for three circuits. The intensity will be at 75–80% of one repetition maximum performing 10 repetitions of each exercise interspersed by 20–30 s to allow for safe movement between exercises. The recovery period between circuits will last 2–3 min. Following each exercise session, patients will undertake a 5 min cool-down period, involving upper-limb light intensity aerobic exercise and some light stretching. Patients will wear heart rate (HR) monitors throughout the exercise sessions. HR and the rating of perceived exertion (RPE) will be assessed at regular intervals throughout the supervised exercise session. The whole duration of each exercise session will be approximately 45 min.

### Clinical management

All the patients in both studies will continue receiving their standard medical treatment, i.e. calcium channel blockers (nifedipine, sildenafil), and will be reviewed in the clinic as often as is deemed clinically necessary. Moreover, in case of adverse events or worsening of symptoms due to exercise or patients request, patients will be withdrawn from the study for health and safety reasons.

### Data management

The study will adhere to the Data Protection Act (1998). Data from this study will be anonymised and stored in password-protected computer systems accessible only by the members of the research team in order to guarantee confidentiality to patients. Paper forms will be stored in locked filing cabinets. All data documents will be kept in a secured and continuously monitored building where the project will be conducted (Collegiate Hall, Collegiate Crescent, Sheffield Hallam University). All study materials will remain in this location for data entry and storage. Double data entry will be implemented to ensure validity and data quality. Participant names will not be used to identify any data. Instead, patients will be assigned a study identification number in order to anonymise their information.

Data will all be anonymised according to the NHS Code of Confidentiality and GMC Good Medical Practice, while publications will not contain identifiable personal data. Furthermore, only research team members will have access to personal patient data during the project, while data will be analysed locally by the study’s statistician. Data will be kept for a 10-year period following the study completion to allow the study team to re-analyse it in case of the development of a new method of analysis and for the data to be used for the development of a future definitive RCT.

### Safety monitoring

We will record all serious adverse events (as defined below), as well as all non-serious adverse events that are deemed to be related to participation in the research, during the period between the provision of informed consent through to 6 months after randomisation.

Serious adverse events are defined as any untoward medical occurrence that either results in death, is life-threatening (i.e. the patient is at risk of death at the time of the event occurring), requires unplanned admissions to emergency hospitalisations or prolonged hospitalisation (deemed to be where a patient’s stay is longer than expected, e.g. patient is operated on as a day case but remains in hospital overnight), results in persistent or significant disability or incapacitation, or results in a congenital abnormality or birth defect.

A non-serious event in the context of this trial will be any untoward medical occurrence to the participant that is related to the patients’ involvement in the study but does not fulfill any of the serious adverse event criteria.

Study investigators will be responsible for recording adverse events and for determining the seriousness, severity, causality and expectedness of any such events. The Chief Investigator (MK) and a delegated clinician (MA) will then be responsible for reviewing all adverse events and confirming that they have been appropriately classified.

A serious adverse event will be reported to the Research Ethics Committee and Sponsor where, in the opinion of the Chief Investigator and the delegated clinician, the event was related to the administration of a research procedure or resulted in death regardless of relatedness.

Patients will be asked to contact the study team to inform them about adverse events if and when they occur. Study investigators will also question patients about the occurrence of adverse events during each participant study visit.

### Outcome measures

The timeline for the outcome measures will be the baseline measurements, and the 3- and 6-month follow-ups after randomisation.

### Feasibility outcomes

The primary aim of this study is acceptability and feasibility of procedures for recruitment, allocation, measurement and retention as well as the adherence and enjoyment levels of the exercise programme. Recruitment rates will be assessed as the rate of invited patients who are eligible and consenting and will be reported in a CONSORT (Consolidated Standards of Reporting Trials) patient flowchart. Suitability of measurement procedures will be assessed via interviews and reasons for missing data. Reasons for dropouts will be used to evaluate the acceptability of allocation procedures and exercise programme. The acceptability of the exercise programme will also be assessed through questionnaires relevant to exercise task self-efficacy, intentions for engagement to exercise, enjoyment levels of exercise and the affect during exercise, and the physiological stress of exercise monitored by HR and RPE. The safety of exercise will be evaluated by the dropout number from the exercise group, and the number and type of adverse events.

### Enjoyment and tolerance of exercise

The feasibility and the perceived enjoyment of HIIT and RT will be assessed through measures that will interpret patients’ perception regarding the (1) exercise intensity, (2) the affect, (3) the exercise task self-efficacy, (4) the intentions and (5) the enjoyment. The above data will be collected at the first and last exercise session each month in order to examine several time points during the exercise intervention. Specifically, the questionnaires will be administered at the 1st, 8th, 9th, 16th, 17th and 24th exercise sessions.

#### Exercise intensity

The RPE will be measured during exercise through a 20-point Borg scale [24] when 2.5%, 8.2%, 42.5%, 48.2%, 92.5% and 98.2% of the exercise programme is completed. These time points have been chosen to incorporate both interval and recovery periods during HIIT. In the RT, the time points will be straight after the completion of each exercise. The 20-point Borg scale ranges from 6 to 20 with anchors ranging from ‘No exertion at all’ (0) to ‘Maximal exertion’ [20]. Apart from measurement at the specified time points during exercise, the RPE will be measured pre- and post-exercise as well as 10 min after the exercise session. Participant’s HR will also be recorded using Polar HR monitors at the same time points as the RPE.

#### Affective valence

The one-item Feeling scale [25] will be used to measure the general affective valence (e.g. pleasure and displeasure) during the exercise session at the same time points as the RPE (Additional file 1). At the beginning of the first exercise session, patients will be provided with the following instructions: “Experiencing alterations in your mood is very common while performing exercise. The sense of pleasure or displeasure varies among individuals during the exercise; in addition, feelings may fluctuate across time. So the answers might feel good and bad a number of times during exercise, when you will be asked to express your feelings using the scale below”. The feeling scale is scored on an 11-point bipolar scale ranging from − 5 to + 5. Seven anchors are provided ranging from ‘Very good’ (+ 5) to ‘Very bad’ (− 5).

#### Exercise task self-efficacy

Patient’s confidence in their ability to repeat the exercise session that they will just have completed will be assessed only after the first exercise session at 20-min post-exercise using a three-item measure (Additional file 2). Each question will include the same introductory theme, “How confident are you that you can…”. The three-items will be (1) “… perform one bout of exercise a week for the next 4 weeks that is just like the one you completed today?”, (2) “… perform two bouts of exercise a week for the next 4 weeks that is just like the one you completed today?” and( 3) “… perform three bouts of exercise a week for the next 4 weeks that is just like the one you completed today?”. The scale score will vary from 0% (Not at all) to 100% (Extremely confident) in 10% increments. The specificity of the three items measure is formed and adapted according to Jung et al. [26].

#### Intentions

Patient’s intentions to engage in the exercise session over the next month will be measured utilising a two-item measure (Additional file 3) at the 1st, 9th and 17th exercise session, 20-min post-exercise [26]. Particularly, patients will be asked “Please rate the extent to which you agree with the following statements (1) ‘I intend to engage in the type of exercise I performed today at least 2 times per week during the next month’ and (2) ‘I intend to engage in the type of exercise I performed today at least 3 times per week during the next month’”. Answers will be scored on a 7-point rating scale with anchors ranging from ‘Very unlikely’ [1] to ‘Very likely’ [7]. The two items will be analysed individually.

#### Enjoyment

Patient’s enjoyment of the assessed exercise sessions will be examined using a modified version of the Physical Activity Enjoyment Scale [27] 20-min post-exercise. This 18-item measure is scored on a 7-point bipolar scale (Additional file 4). Example items are “I find it energising/I find it tiring” and “it’s very pleasant/it’s very unpleasant”. The original measure is amended by erasing one of the 18 items that is irrelevant due to the time point that will be measured (“I am absorbed in the activity/I am not at all absorbed in the activity”). Moreover, the original Physical Activity Enjoyment Scale instructions are amended from “Please rate how you feel AT THE MOMENT about the physical activity you have been doing” to “Please rate how you feel about the exercise you just completed”. Both modifications were made to reflect the correct time point at which the questions/questionnaire will be administered (20-min post-exercise).

### Patient experiences for study procedures

We aim to undertake an in-depth exploration of the patients’ study experience in a subsample of six patients from each group (exercise and control group). Interviews lasting between 30 and 35 min will take place 3 months after randomisation for both groups.

We aim to explore (1) patients’ experiences of RP; (2) experiences of treatment and advice received pre FESS trial; (3) participant’s preference for trial allocation (exercise or control group); (4) experiences of study participation in both the exercise intervention group and control group; and (5) participant’s acceptability of the exercise intervention and study procedures.

We will adopt a constructivist approach [28], which recognises the individual and personal nature of a patient’s exercise experiences both before and during the trial. Semi-structured face-to-face in-depth interviews will be conducted at the Centre for Sports and Exercise Science at Sheffield Hallam University in a comfortable and private room. Interviews will be recorded and then transcribed verbatim, and will then be analysed thematically by using framework analysis (familiarisation, identifying a thematic framework, indexing, charting and mapping).

Sample and recruitment

Six and six patients from exercise and control group, respectively, will be recruited using purposive sampling (mixture of sexes, younger and older patients from exercise and control group).

### Secondary outcomes

#### Digital cutaneous microvascular function

##### Iontophoresis

Microvascular assessments using LDF and iontophoresis (as described in the literature; [22]) will be performed in a temperature-controlled room (22–24 °C). LDF electrodes will be attached to the dorsal aspect of the reference fingers for acetylcholine (ACh) and sodium nitroprusside (SNP) administration. These will be used as indicators of the changes occurring in the endothelial (dependent and independent) vasodilatory function. HR (Sports Tester, Polar, Finland) and blood pressure of the brachial artery (left arm; Dinamap Dash 2500, GE Healthcare, USA) will be monitored at 5-min intervals throughout the protocol. The two drug delivery electrodes (PF383; Perimed AB, Jarfalla, Sweden) will be positioned over the healthy-looking skin, approximately 4 cm apart with one containing 100 μL of 1% ACh (Miochol-E, Novartis, Stein) and the other 80 μL of 1% SNP (Nitroprussiat, Rottapharm). A battery-powered iontophoresis controller (PeriIont PF382b; Perimed AB) will be used to provide the charge needed for ACh and SNP delivery. A 4 min stable recording of baseline flux will be followed by administration of the two agents according to the following protocol: 0.2 mA for 10 s (i.e. 2 mC), 0.2 mA for 15 s (i.e. 3 mC), 0.2 mA for 20 s (i.e. 4 mC), and 0.3 mA for 20 s (i.e. 6 mC), occurring between 4-min intervals [21, 22]. To obtain an index of skin blood flow, cutaneous red cell flux will be measured by placing an iontophoresis laser Doppler probe (PF481–1; Perimed AB), connected to a laser Doppler fluximeter (PF5001; Perimed AB).

##### Peak oxygen uptake test

During the cardiopulmonary tests, gas exchange will be collected and analysed by an online breath-by-breath analysis system (Ultima™, Medical Graphics, UK). The gas analyser will be calibrated before each test according to the manufacturer’s calibration guidelines. HR breathing frequency, tidal volume, minute ventilation, VO_2_ and volume of exhaled carbon dioxide, and their ratios, as well as the respiratory exchange ratio, will be displayed on a monitor (BreezeSuite, MGC Diagnostics, USA) on a breath-by-breath analysis. HR will be continuously monitored using a Polar HR monitor (Polar FS1, Polar Electro, Kemple, Finland) and blood pressure will be assessed by the researcher using a manual sphygmomanometer (DuraShock DS54, Welch Allyn, USA) and a stethoscope (Littman Classic II, 3 M, USA). The RPE will be recorded during the last 10 s of every minute during the exercise test until volitional exhaustion using Borg’s scale [24] with 6–20 points. Peak power output and test duration will be measured in both tests. VO_2peak_ will be defined as the average oxygen consumption recorded from expiratory samples during the final 30 s of exercise.

##### Arm crank ergometer protocol

The arm crank ergometer will be adjusted to ensure alignment between the ergometer’s crankshaft and the centre of the glenohumeral joint. The patient’s sitting position will also ensure that the elbows are slightly bent when the arm is outstretched and will be instructed to maintain their feet flat on the floor at all times. Due to different power capabilities, two different protocols will be instructed for men and women. Men will commence at a workload of 40 W and women at 20 W. In both protocols, the crank rate will be maintained at 70 rev min^− 1^ [29] and the linear ramp increments will be increased by 1 W/6 s and 1 W/10 s for men and women, respectively [30]. The test will commence with 2 min resting and 2 min of warm-up unloaded. A RPE of 18 or above and/or the inability to maintain a crank rate above 60 rev min^–1^, or if any contraindication signs emerge [31] will result in the termination of the test. Following termination of the exercise part of the test, an unloaded exercise will be performed for 2–3 min allowing an active cool down.

#### Transcutaneous oxygen pressure (TcpO_2_)

TcpO_2_ measurements will be performed during the cardiorespiratory tests using sensors that will be non-invasively attached to the skin and allow to heat. The sensors induce skin blood capillary dilatation through heat, which increases the blood flow and results in oxygen diffusion through the skin to the sensor. The sensor measures TcpO_2_ values inwardly through an electrochemical process.

Measurements will be performed using the TINA TCM400 TcpO_2_ device (Radiometer, Copenhagen, Denmark). The temperature of the probe will be set to 44.5 °C to allow maximal skin vasodilation, thereby decreasing the arterial-to-skin surface oxygen pressure gradient. Before the exercise test, 15–20 min will be allowed with the probe attached to the skin for stabilisation of TcpO_2_ value. Following the test, the TcpO_2_ values will be automatically corrected according to a temperature of 37 °C by the TINA device. The electrode will be placed slightly below the right scapula on the back away from any bone.

Fixation rings will be used to hold the probe attached to the skin and this will be filled with two small drops of contact fluid before attachment to the sensor. The fluid will then be heated causing the subsequent dilatation of the skin. The raw values of the patient’s oxygen perfusion will be defined as previously described in Wasilewski et al. [32].

#### Health-related quality of life (HRQoL)

##### EQ-5D-5L questionnaire

The EQ-5D-5L questionnaire will measure the main outcome to assess the patients’ HRQoL throughout the study (baseline, 3- and 6-month follow-up). The EQ-5D-5L is a generic measure of health status, where health is characterised by five dimensions (mobility, self-care, ability to undertake usual activities, pain, anxiety/depression) [19]. Patients will be asked to describe their level of health on each dimension using one of five levels, namely no problems, slight problems, moderate problems, severe problems or extreme problems. To evaluate the EQ-5D-5L outcome in the overall pre- and post-exercise intervention we will calculate the quality-adjusted life years (a metric used in cost-utility analysis that combines length and quality of life in a single parameter; [33]). We will also report digital ulcers, hospitalisation for iloprost infusion and digital amputations.

##### Functional ability test

The functional ability will be assessed through a 6-minute walking test. Although this test lacks organ specificity in SSc, it can provide a valuable outcome parameter and, thus, is suggested as a regular assessment in this clinical condition [23]. Patients will be instructed to walk as far as possible back and forth on a 10 m corridor for 6 minutes. They will also be instructed to slow down, stop and/or rest as necessary if they experience breathlessness or become exhausted, but to resume walking as soon as they feel able to. The laps and the total walking distance will be recorded on a worksheet.

### Statistical analysis

The sample size for a feasibility study should be adequate to estimate the critical metrics needed to assess the feasibility of conducting the definitive study, with sufficient precision [34]. The critical metrics are the consent rate (i.e. the proportion of eligible patients who consent to participate and be randomised), improvement in microcirculation and quality of life as well as compliance with treatment, and attrition rates. Fifteen patients in each group (*n* = 30 in total) will provide a sufficiently precise (within 15 percentage points for a 90% confidence interval) estimate of the proportion willing to be randomised, assuming 35–40% intention to be randomised.

The SPSS software (version 23, IBM SPSS, New York, USA) will be used to enter quantitative data and cleaning the data and to carry out missing values imputation and statistical analysis. The descriptive statistics, including mean and standard deviation, will be presented for basic demographics and primary and secondary outcomes of the patients in intervention and control groups. For categorical data, the χ^2^ test will be used to infer on the significant association. Independent and paired samples *t* tests will be used to account for the changes upon the primary and secondary outcomes within the group at pre- and post-exercise intervention but also between the two groups. Kolmogorov–Smirnov and Shapiro–Wilk tests will be used to test the normal distribution of the dependent variable and Levene’s test will be used to test the homogeneity of variances. Pearson’s r will test any relations and interactions between our variables. The qualitative analysis will be performed using framework analysis [35], aiming to describe individuals’ experiences of exercise, searching for common, recurrent patterns but also exploring participant experiences that might explain behaviour and improve advice and services in the future. Data saturation will be achieved when similar themes arise repeatedly and no new themes arise in the interview.

## Discussion

Digital ischemia is a direct result of RP in SSc patients that adversely affects their HRQoL. The number of incidents that require hospitalisation and, occasionally, digital amputation is not negligible (30%) [2]. There is strong evidence from a wide range of clinical conditions in which vascular disease plays a leading role in their pathophysiology, that exercise, and more specifically HIIT and RT, are able to improve the endothelial vascular function and, thus, to increase macro- and microcirculation. Based on that evidence, we will attempt to implement a combined exercise protocol, primarily assessing its feasibility and, secondarily, its efficacy on vascular tone. The feasibility study will provide important evidence about the effectiveness of supervised exercise training as a complementary treatment to the usual care (e.g. medical treatment) for improving microcirculation in the digital area, physical fitness, functional ability and HRQoL in SSc patients. The findings will inform a future definitive multicenter randomised controlled trial with a larger sample size. We acknowledge that our sample size might be a limitation for the current study but we need to stress that FESS is a feasibility study in a novel area and we will strictly adhere to the pre-defined eligibility criteria to present a consistent and reproducible outcome. Moreover, the ratio between women and men is uneven; however, the women to men ratio in those with SSc is estimated to 5.2:1 in northeast England [36]. Overall, the study is expected to provide valuable evidence for future research with SSc patients.

## Trial status

Recruitment started in January 2018 and is ongoing.

## Additional files

Additional file 1: Feeling scale. (DOCX 35 kb)

Additional file 2: Exercise task self-efficacy. (DOCX 37 kb)

Additional file 3: Intentions for engagement to exercise. (DOCX 34 kb)

Additional file 4: Physical activity enjoyment scale. (DOCX 44 kb)

## Abbreviations

ACh: acetylcholine
FESS: Feasibility of Exercise in Systemic Sclerosis
HIIT: high-intensity interval training
HR: heart rate
HRQoL: quality of life
LDF: laser Doppler fluximetry
RP: Raynaud’s phenomenon
RPE: rating of perceived exertion
SNP: sodium nitroprusside
SSc: systemic sclerosis
TcpO_2_: transcutaneous oxygen pressure
VO_2peak_: peak oxygen uptake
