# Supplementary material for: Investigating the effectiveness and feasibility of exercise on microvascular reactivity and quality of life in systemic sclerosis patients: study protocol for a feasibility study
Source: Trials. 2018 Nov 21;19:647. doi: 10.1186/s13063-018-2980-1 (PMC6249907; doi:10.1186/s13063-018-2980-1)
Supplement: Supplementary file 3 — Intentions for engagement to exercise. (DOCX 34 kb) [file 13063_2018_2980_MOESM3_ESM.docx]

**Appendix C**

**Intentions for engagement to exercise**

Please rate the extent to which you agree with the following statements.

1) I intend to engage in the type of exercise I performed today at least 2 times per week during the next month.

| 1 | 2 | 3 | 4 | 5 | 6 | 7 |
| --- | --- | --- | --- | --- | --- | --- |
| Very unlikely | Unlikely | Slight unlikely | Neutral | Slight likely | Likely | Very likely |

2) I intend to engage in the type of exercise I performed today at least 3 times per week during the next month.

| 1 | 2 | 3 | 4 | 5 | 6 | 7 |
| --- | --- | --- | --- | --- | --- | --- |
| Very unlikely | Unlikely | Slight unlikely | Neutral | Slight likely | Likely | Very likely |
