# Supplementary material for: Investigating the effectiveness and feasibility of exercise on microvascular reactivity and quality of life in systemic sclerosis patients: study protocol for a feasibility study
Source: Trials. 2018 Nov 21;19:647. doi: 10.1186/s13063-018-2980-1 (PMC6249907; doi:10.1186/s13063-018-2980-1)
Supplement: Supplementary file 4 — Physical activity enjoyment scale. (DOCX 44 kb) [file 13063_2018_2980_MOESM4_ESM.docx]

**Appendix D**

**Physical Activity Enjoyment Scale**

Please rate how you feel about the exercise you just completed.

Look at the statement, choose the left or right hand side of the statement that **most** represents how you felt about participating in your recent exercise session, then choose a number on your chosen side to show how much you agree with that statement.

 If you don’t agree with either side of the statement choose neutral (4).

 Only circle one number per statement.

 Example: I enjoyed it or I hated it.

 If you mainly enjoyed the exercise session you would circle 2.

|  | Absolutely  agree | Mainly  agree | Somewhat  agree | Neutral | Somewhat  agree | Mainly agree | Absolutely agree |  | |
| --- | --- | --- | --- | --- | --- | --- | --- | --- | --- |
|  | 1 | 2 | 3 | 4 | 5 | 6 | 7 |  |  |
| I enjoyed it |  |  |  |  |  |  |  | **I hated it** |  |
|  | 1 | 2 | 3 | 4 | 5 | 6 | 7 |  |  |
| I felt bored |  |  |  |  |  |  |  | **I felt interested** |  |
|  | 1 | 2 | 3 | 4 | 5 | 6 | 7 |  |  |
| I disliked it |  |  |  |  |  |  |  | **I liked it** |  |
|  | 1 | 2 | 3 | 4 | 5 | 6 | 7 |  |  |
| I found it pleasurable |  |  |  |  |  |  |  | **I found it unpleasurable** |  |
|  | 1 | 2 | 3 | 4 | 5 | 6 | 7 |  |  |
| It was not fun at all |  |  |  |  |  |  |  | **It was a lot of fun** |  |
|  | 1 | 2 | 3 | 4 | 5 | 6 | 7 |  |  |
| I found it energizing |  |  |  |  |  |  |  | **I found it tiring** |  |
|  | 1 | 2 | 3 | 4 | 5 | 6 | 7 |  |  |
| It made me depressed |  |  |  |  |  |  |  | **It made me happy** |  |
|  | 1 | 2 | 3 | 4 | 5 | 6 | 7 |  |  |
| It was very pleasant |  |  |  |  |  |  |  | **It was very unpleasant** |  |
|  | **Absolutely**  **agree** | **Mainly**  **agree** | **Somewhat**  **agree** | **Neutral** | **Somewhat**  **agree** | **Mainly agree** | **Absolutely agree** |  | |
|  | 1 | 2 | 3 | 4 | 5 | 6 | 7 |  |  |
| I felt good physically while doing it |  |  |  |  |  |  |  | **I felt bad physically while doing it** |  |
|  | 1 | 2 | 3 | 4 | 5 | 6 | 7 |  |  |
| It was very invigorating |  |  |  |  |  |  |  | **It was not at all invigorating** |  |
|  | 1 | 2 | 3 | 4 | 5 | 6 | 7 |  |  |
| I was very frustrated by it |  |  |  |  |  |  |  | **I was not at all frustrated by it** |  |
|  | 1 | 2 | 3 | 4 | 5 | 6 | 7 |  |  |
| It was very gratifying |  |  |  |  |  |  |  | **It was not at all gratifying** |  |
|  | 1 | 2 | 3 | 4 | 5 | 6 | 7 |  |  |
| It was very exhilarating |  |  |  |  |  |  |  | **It was not at all exhilarating** |  |
|  | 1 | 2 | 3 | 4 | 5 | 6 | 7 |  |  |
| It was not at all stimulating |  |  |  |  |  |  |  | **It was very stimulating** |  |
|  | 1 | 2 | 3 | 4 | 5 | 6 | 7 |  |  |
| It gave me a strong sense of accomplishment |  |  |  |  |  |  |  | **It did not give any sense of accomplishment** |  |
|  | 1 | 2 | 3 | 4 | 5 | 6 | 7 |  |  |
| It was very refreshing |  |  |  |  |  |  |  | **It was not at all refreshing** |  |
|  | 1 | 2 | 3 | 4 | 5 | 6 | 7 |  |  |
| I felt as though I would rather be doing something else |  |  |  |  |  |  |  | **I felt as though there was nothing else I would rather doing** |  |
